# One-stage multiple root canal treatment of adjacent teeth combined with surgical apicectomies may be preferred in patients with severe anxiety under local anaesthesia: a case report

**DOI:** 10.1186/1757-1626-1-262

**Published:** 2008-10-23

**Authors:** Panagiotis Kafas, Christos Stavrianos, Georgios Kafas

**Affiliations:** 1 Department of Oral Surgery and Radiology, School of Dentistry, Aristotle University, Thessalonica, Greece; 2 Department of Endodontics, School of Dentistry, Aristotle University, Thessalonica, Greece; 3 Department of Psychiatry, Royal Free Hospital, NHS Trust, London, UK

## Abstract

A female patient referred to the clinic for assessment of a unilateral painful swelling formation on the left anterior maxilla. Anxiety is a psychological condition commonly seen in dental practice. When this psychological alteration affects the patient severely, any dental operation may be found difficult under local anaesthesia. We report a case of one-stage management in a patient with periapical lesion and anxiety.

## Background

Dental anxiety is a specific term describing the altered psychological status of patients visiting dental clinics. Anxiety is a popular term describing the feeling of nervousness or worries about something, and can be characterized by agitation and a diffuse sense of dread [1].

In general, when the patient is fully cooperative, any dental procedure can be performed properly. The eagerness of the dentist to apply his knowledge in the practical field is not the only essential requirement in dentistry under local anaesthesia. The willingness of the patient seems to be equally important as a calm patient is usually fully cooperative [2]. The management of anxious patients may include local anaesthesia with sedation, or general anaesthesia [3]. This depends on the preference and systemic status of the patient, but as a rule, less dental visits may be more easily accepted by the patient.

## Case report

A 47-years-old female patient visited the dental clinic for an evaluation of a painful left maxillary swelling. At the time, she worked in a public company. Apart from various restorative and prosthetic treatments, the dental history was free. The medical history revealed the presence of short-term anxiety without insomnia, atrophic gastritis and irritable bowel. At the time, she was taking diazepam (2 mg, bd, per os). Furthermore, a dietician and gastroenterologist examine her regularly to prevent gastrointestinal complications. No allergies reported. She did not smoke or drink alcohol.

**Figure 1:**
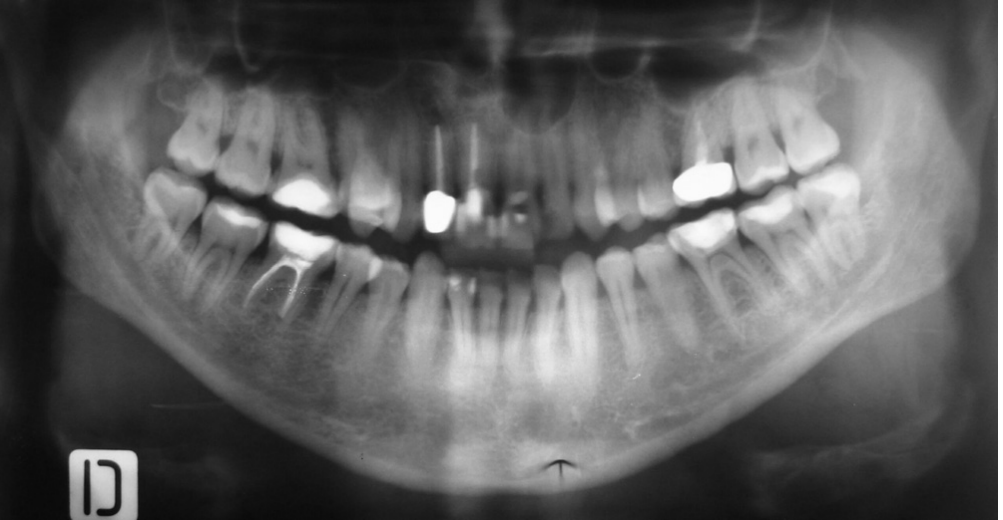
Panoramic tomography revealed the periapical lesion associated with teeth 21,22.

The clinical examination revealed signs of inflammation over the maxillary mucosa. Inspection of the other sides of the oral cavity was found normal. The panoramic tomography revealed endodontic treatments of 11, 12, 26, 46, and conservative or restorative treatments of 11, 12, 16, 26, 27, 36, 37, 38, 42, 44, 46, and 47. In addition to the previous findings, a large periapical radiolucency associated with the non-vital teeth 21,22 was observed (Figure. 1).

**Figure 2:**
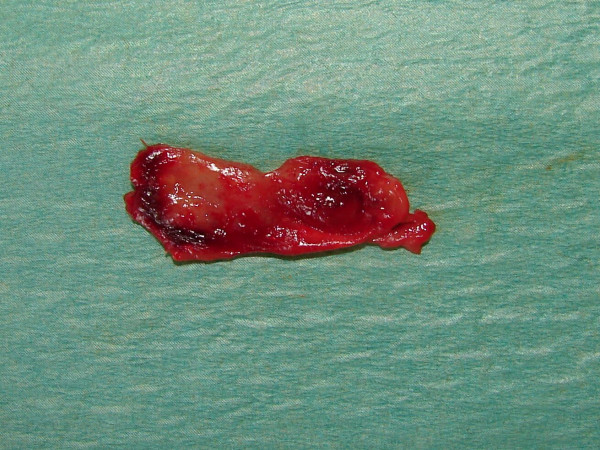
The enucleated periapical lesion.

**Figure 3:**
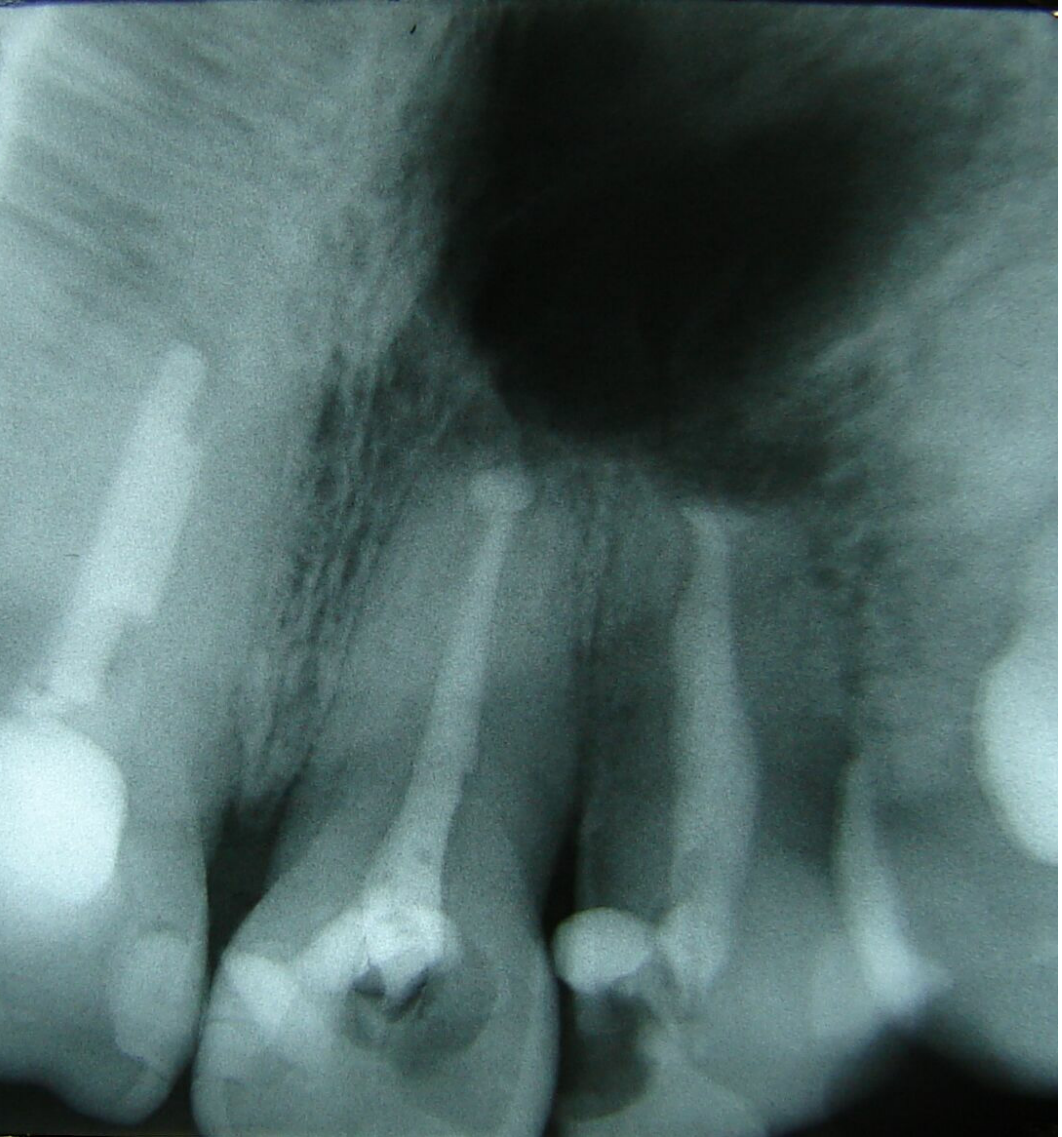
Periapical x-ray showed the filled root canals with the retrograde filling material.

Two ampoules of local anaesthesia (2% lignocaine with 1:80000 adrenaline) were infiltrated on the buccal and palatal mucosa. The broad-based mucoperiosteal flap was raised buccally to expose the area of the periapical lesion. Using a small size round bur in a straight handpiece 1:1 at 28000 rpm allowed the surgical design of the bone "window" over the periapical lesion. The two apices were cut vertically (about 3 mm) to the longitudinal axis of the roots using small-fissured burs to avoid bevelled surfaces. The periapical lesion was enucleated (Figure. 2) with the two apices and stored in 10% buffered formalin. The canals in apices were widened using specially curved diamond-coated ultrasonic tips. The orthograde root canal treatment was performed easily using mechanical rotation NiTi system, 0.12% chlorhexidine digluconate irrigation, diode laser cleaning of the root canals (300 μm fiber, 1000 mW, 808 nm) and Zinc-Oxide Eugenol filling material with laterally condensed gutta-percha cones. This allowed the direct inspection of the instruments passing through the cut apices from the bone opening. The filling material passed through the apices was cleaned and a similar retrograde filling was placed on apical opening to seal the tooth roots (Figure. 3). The flap was sutured using polyglactin-910. A subscription was given to the patient including clindamycin 300 mg, bd, 4/7 and ibuprofen 400 mg, tds, 4/7. The diagnosis of radicular cyst was confirmed histo-pathologically. Three months later the patient was free of any symptoms and signs with optimum tissue healing.

The decision to perform one-stage treatment was compatible to the patient's choice. The patient verbally informed and signed the consent form. The time required for the combined treatment was about an hour.

## Discussion

Anxiety in dentistry is an important limitation in performing various conservative and surgical dental procedures [2]. One recommendation that may solve this situation is to either perform dental operations under local anaesthesia combined with sedation or to employ general anaesthesia [3].

We have to keep in mind that some dental procedures should be performed in multiple dental visits. Therefore, the multiple application of sedation or general anaesthesia is not suggested. This case report emphasizes the importance of one-stage treatment of a complicated situation, which would otherwise require multiple conservative dental operations, such as root canal treatment, fillings and long-term follow-ups. The value of this procedure is more intense considering the psychological status of the patient. Is it better to manage oral diseases in one stage or more? In our opinion, if the outcome can be the same, the answer is positively one-stage.

This case report also emphasizes the need for one-stage treatment even in cases of combined conventional and surgical management. It was decided to perform root canal treatment of the non-vital left central and lateral upper incisor with surgical excision of the periapical lesion including apicectomies and retrograde apical fillings. In our estimation, the time required for this one-stage treatment is less if compared to the classical method of root canal treatment and surgical apicectomies in multiple dental visits. Furthermore, the above-mentioned one-stage management of such cases under local anaesthesia was found to be convenient for the patient.

Concluding, any medical solution given in less visits, when the outcome is almost the same, could be preferable for patients with anxiety. Furthermore, the one-stage procedure can be more easily performed under general anaesthesia without requiring excessive anaesthetic, as seen in short-term and multiple general anaesthesia surgical procedures. In non-anxious patients the guidelines currently indicate the conservative treatment, such as root canal treatment even in cases of large periapical lesions, as first choice [4]. Surgical solution of inflammatory periapical lesions is only indicated if the conservative treatment fails [5].

## Competing interests

The authors declare that they have no competing interests.

## Authors' contributions

GK and CS analyzed the patient data. PK was major contributor in assessing the case and writing the manuscript. All authors read and approved the final manuscript.

## Consent

Written informed consent was obtained from the patient for the publication of this case report and accompanying images. A copy of the written consent is available for review by the Editor-in-Chief of this journal.
